# Supplementary material for: Hemodynamic profiles by non-invasive monitoring of cardiac index and vascular tone in acute heart failure patients in the emergency department: External validation and clinical outcomes
Source: PLoS One. 2022 Mar 31;17(3):e0265895. doi: 10.1371/journal.pone.0265895 (PMC8970400; doi:10.1371/journal.pone.0265895)
Supplement: S1 File — I—Further methodological detail on consensus clustering, and contrast to k-means cluster analysis. II—Consensus Clustering Dendrograms. III—Delta-area under the cumulative distribution function (CDF) for each additional level K in consensus clustering. IV—Sensitivity Analyses—Methods, Goals, and Rationale. (DOCX) [file pone.0265895.s001.docx]

**Supplement / Supporting Material S1: Supplemental methods**

**I. Further methodological detail on consensus clustering, and contrast to k-means cluster analysis**

The prior study used k-means clustering to derive three hemodynamic profiles among the PREMIUM registry’s AHF patients (derivation cohort, DC). K-means clustering has two major weaknesses: 1. the data analyst must decide before clustering how many clusters (k) to subgroup the data by, and 2. the clustering is a direct function of the data and therefore does not guarantee clusters can be replicated in an external sample. Both weaknesses have the potential to lead to model overfitting. Consensus clustering is a machine learning technique which improves on k-means cluster analysis primarily by addressing these two weaknesses, and thereby reducing the chance of an overfit model^18^. First, the technique does not assume 3 clusters (or any other number) to be the number of truly distinct hemodynamic profiles present. Rather than being pre-specified, the number of distinct hemodynamic profiles to group observations into is iteratively tested for every possible number of clusters 1-10. Second, clustering is replicated 1000 times on random sampling of the dataset after introducing random perturbations of data points, which decreases the potential for bias related to the sampling of the cohort itself. At each number of clusters tested (1-10), the resampled cluster analyses are pooled to arrive to a “consensus” score wherein patients are grouped to maximize similarity in cardiac index and vascular tone within a cluster, while maximizing differences for patients in different clusters. An elbow plot of the delta area change in cumulative distribution function and other diagnostics are then produced which indicate the point at which splitting the data into additional clusters no longer enhances within-profile similarity or between profile differences for CI and SVRI (i.e. the point past which further subdivisions/higher K reclassifies data by random/meaningless divisions). For further methodological details on consensus clustering, see Wilkerson et al.^40^. For consensus clustering in the validation cohort (VC) for this study, cardiac index and vascular tone were log transformed and scaled to facilitate efficient clustering by Euclidean distance.

**II: Consensus Clustering Dendrograms**

Consensus Clustering^40^ expands on K-means cluster analysis in two primary ways: 1. Repeating clustering with random perturbations of the data to internally-validate cluster stability, and 2. Assessing clustering at multiple levels k (k = number of times the data is divided). Dendrograms below, for each k tested in the dataset (2-10), show the proportion of repeated clustering runs in which each segment of the data was assigned to a given cluster. Dark blue indicates a data segment which is always assigned to the same cluster (i.e. maximum consensus/cluster stability) while lighter blues indicate where a data segment was fit into one of multiple clusters based on the random data perturbations (low consensus/cluster stability). A lack of consensus/cluster stability suggests that clustering is more due to random chance than true similarity within the data clusters.

In the dendrograms (this page and next), cluster stability appears to reach a maximum at k=3, with nearly random clustering for k>5. Thus, a choice of 3 groups (3 divisions of the data) appears to maximize within cluster similarity and maximize between-cluster differences.


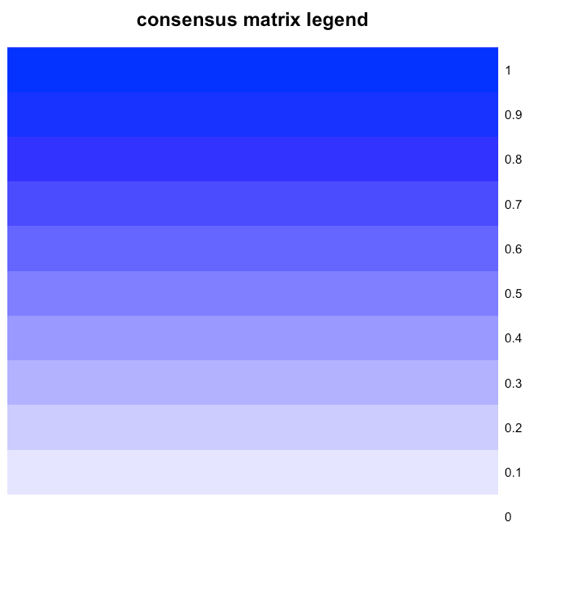

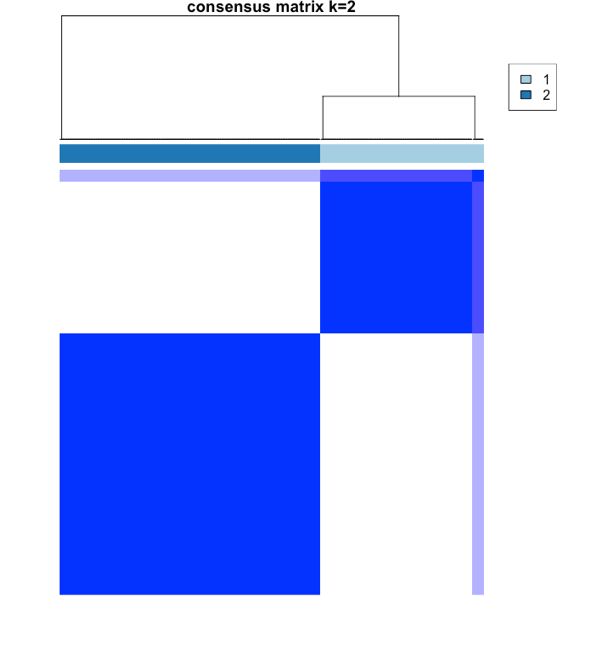

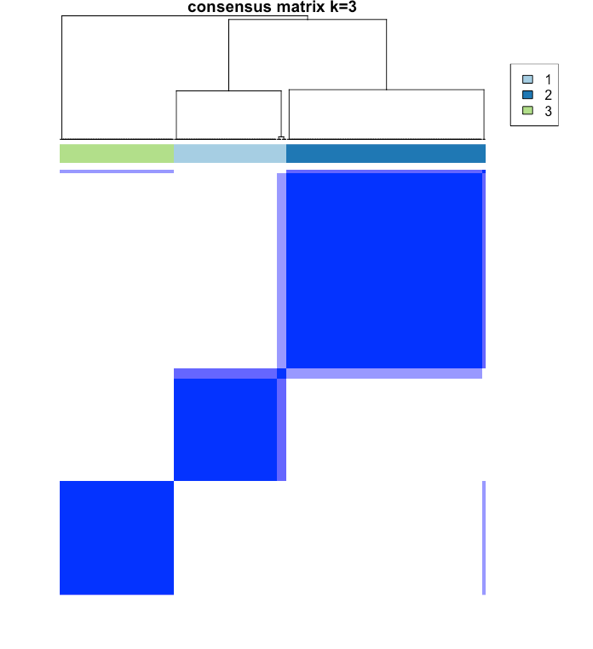

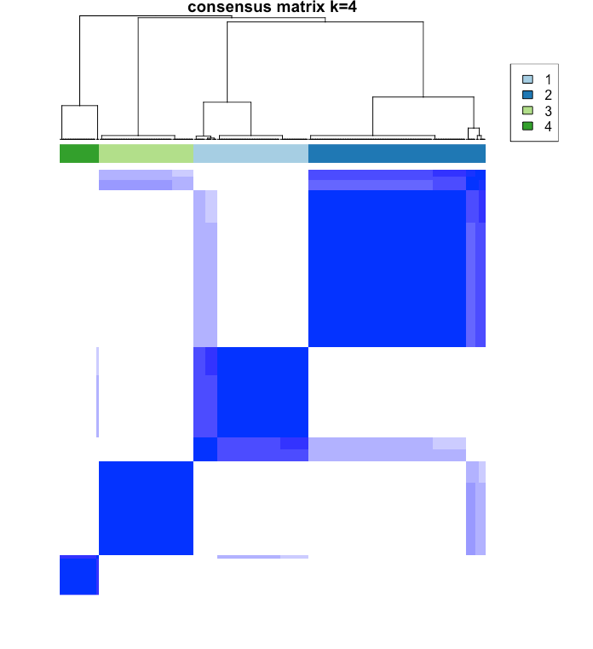

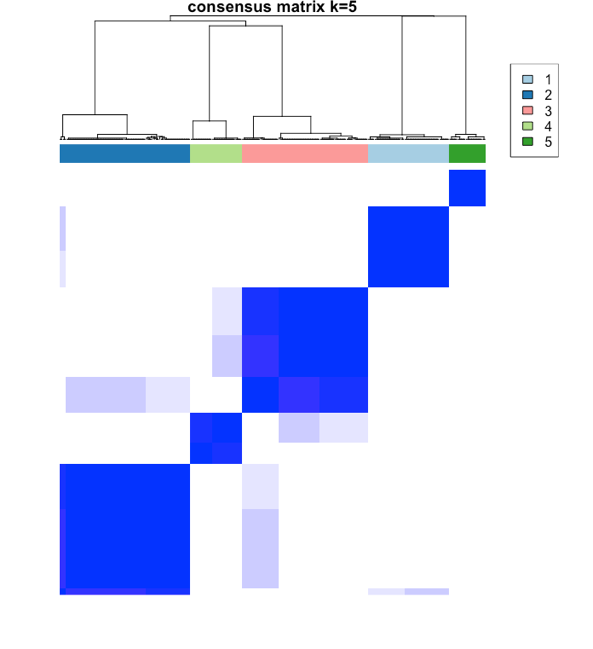

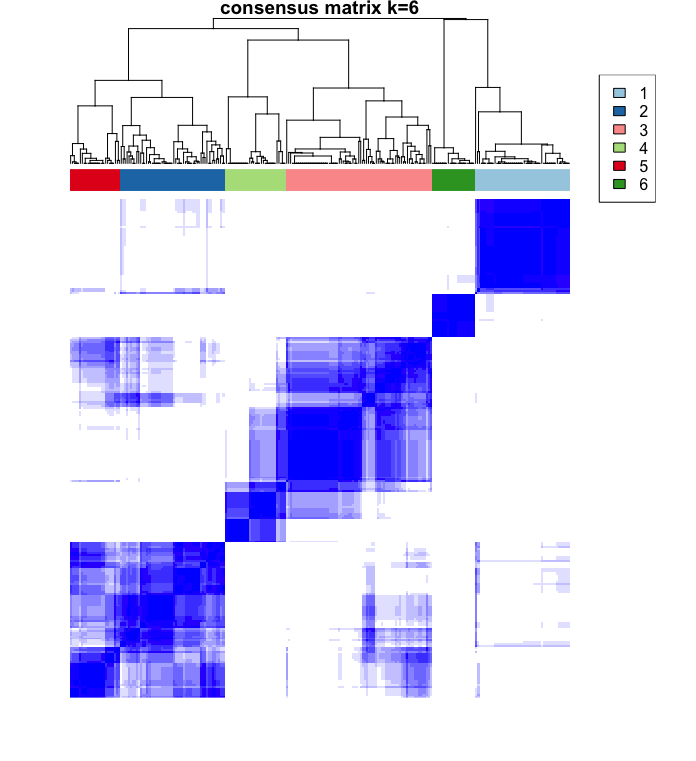

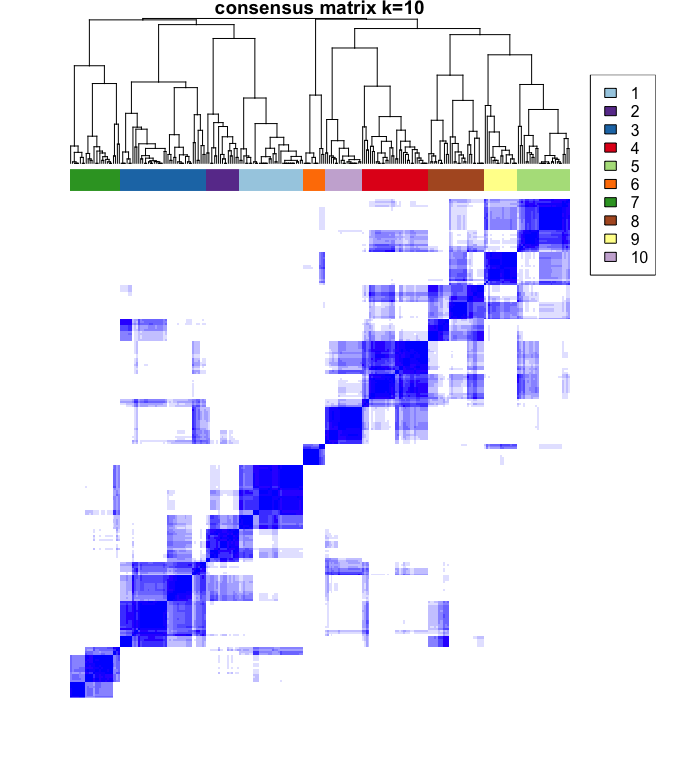

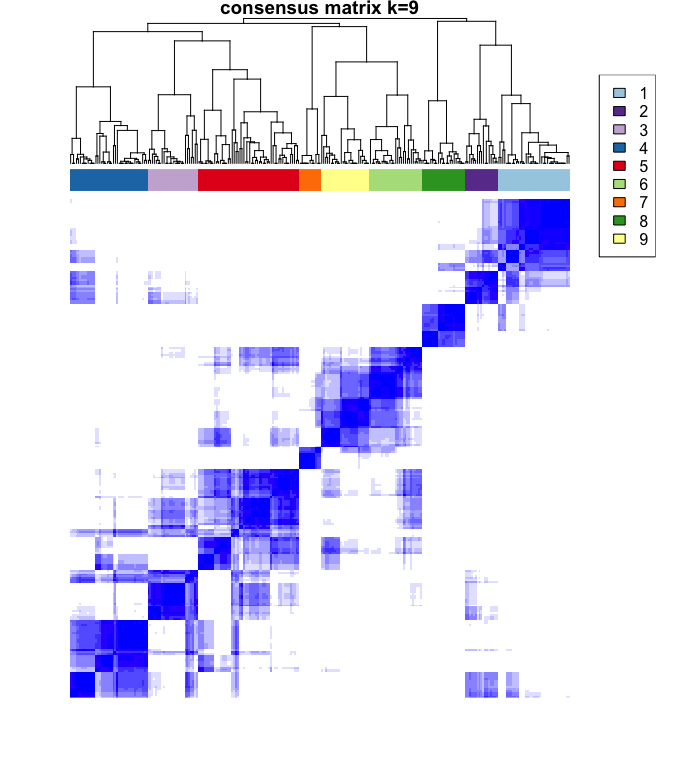

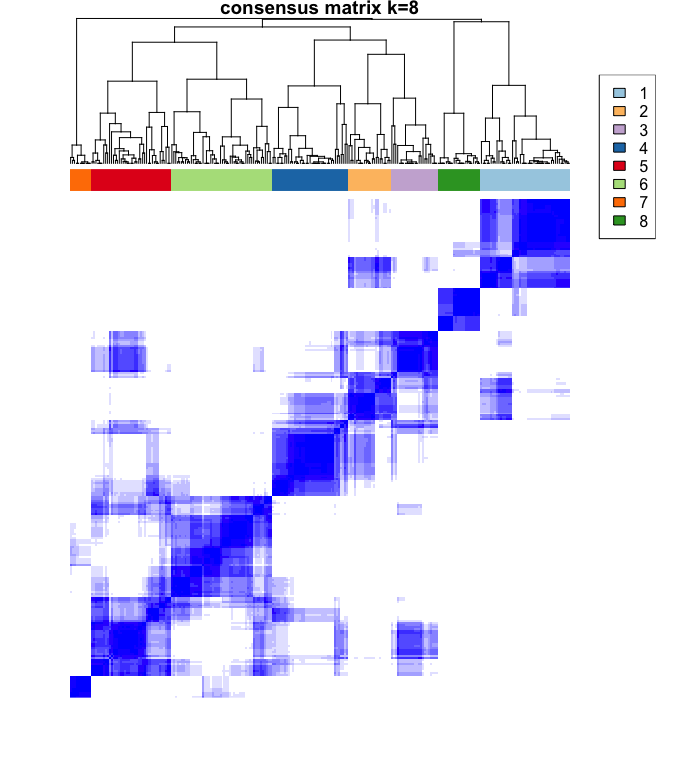

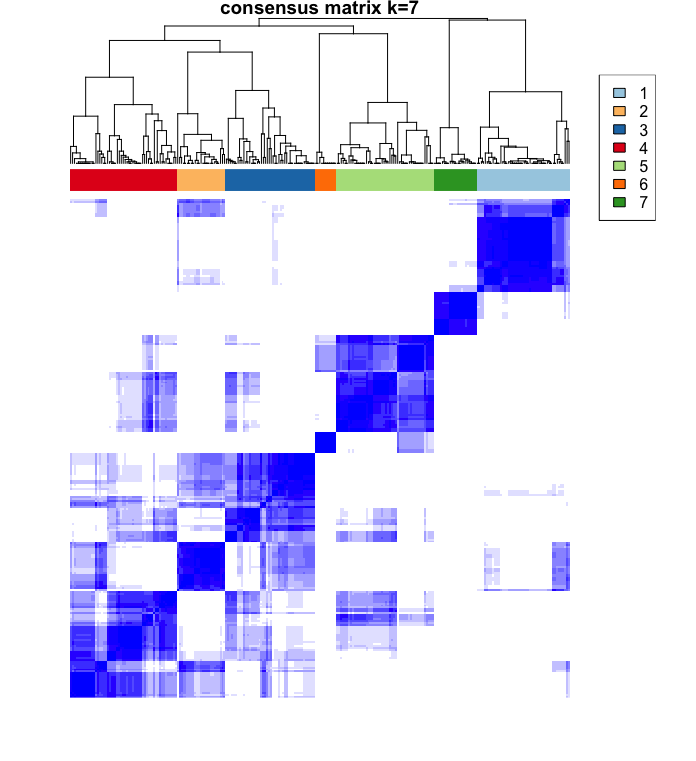


**III: Delta-area under the cumulative distribution function (CDF) for each additional level K in consensus clustering**

In this elbow plot, the relative change in consensus CDF is shown for each additional data division (i.e. 1 increment increase in k) during consensus clustering. The elbow, where further increases in k yield relatively little change in area under the CDF curve, indicates the K at which further additional clusters add little to the goal of class discovery. The elbow occurs around k=4 in the validation cohort. This further supports what is suggested in supplemental figure S2: additional divisions of the data beyond k=3 yields little improvement in class discovery and stability.


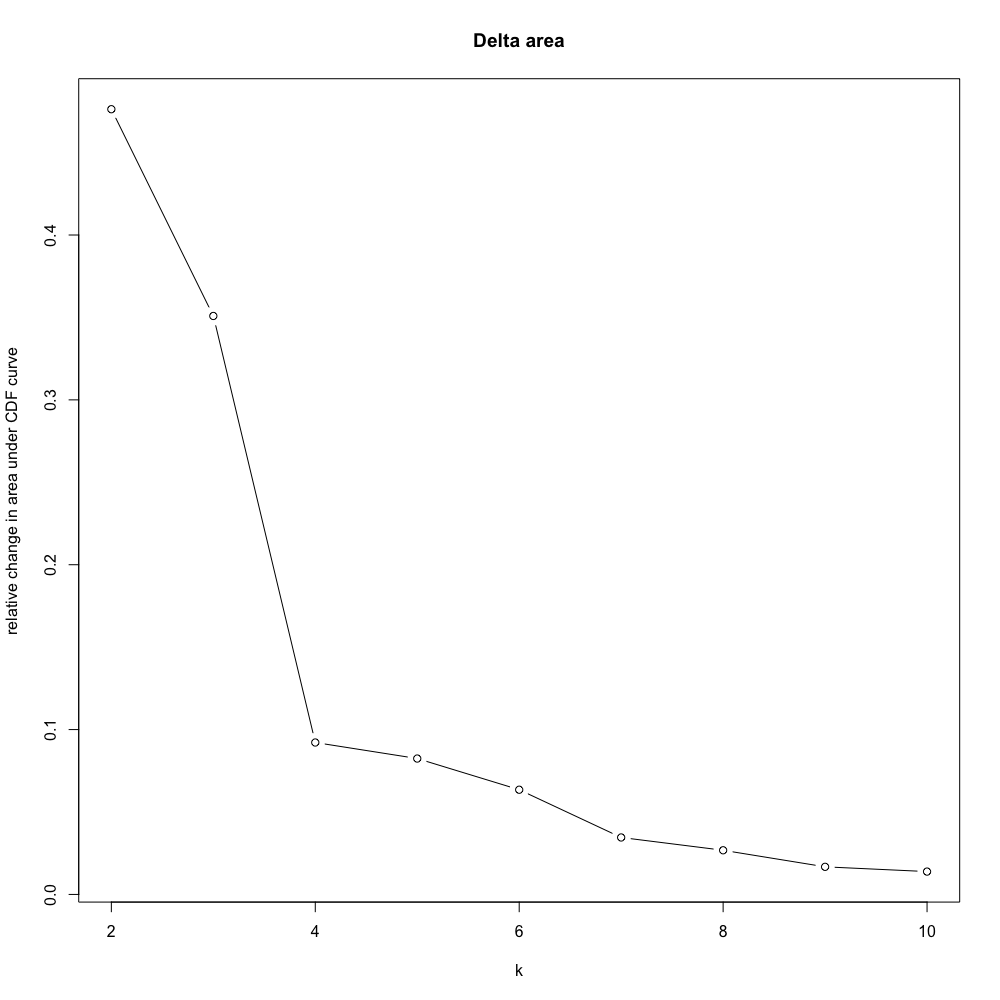


**IV: Sensitivity Analyses - Methods, Goals, and Rationale**

**Sensitivity analysis 1: Validation cohort hemodynamic profile as unique novel parameter versus being a simple combination of conventional clinical features**

Principal components analysis (PCA), stratified by VC hemodynamic profile, was performed including all variables collected. If between-profile differences could be easily explained by more conventional clinical variables, then relatively few variables should account for a large amount (>75%) of between-profile variance. We reasoned that clinicians likely could not intuit the same information provided by the profiles if it required >3-4 variables to explain the single profile variable.



**Sensitivity analysis 2: differences between validation cohort profiles in clinical markers of instability and/or severity**

VC hemodynamic profiles were compared by the observed (actual) ED discharge rate in the sample, and by clinical features which would suggest to the EP an obvious need for hospital admission (i.e. potential indications of a patient not being safe for ED discharge). Based on local practice patterns, we considered a clear indication for admission as any critical care intervention in the ED (positive pressure ventilation (PPV), inotropes, IV vasodilators, IV rate control for tachydysrhythmia) or unstable vital signs (respiratory rate > 30 or < 6, heart rate >120 or < 50, SBP >200 or < 90, SpO2 < 88% or requiring supplemental oxygen).

**Sensitivity analysis 3: Differences between validation cohort patient profiles and profiling of concurrently enrolled patients adjudicated to not have acute heart failure**

Patients who met all inclusion and exclusion criteria for the VC, but were later adjudicated not to have AHF (i.e. those with non-cardiac dyspnea and/or chronic heart failure without an acute decompensation) had the same data collected as those included in the study (including hemodynamic monitoring). This internal group was used as a second control cohort (“CC2”), under the hypothesis that (like the septic patients in the external CC) they should have different profiling of hemodynamics than those in the VC. CC2 patients were profiled and compared to VC profiles by similar methods as the CC (consensus clustering, followed by PERMANOVA).
